# Melatonin and Phytomelatonin: Chemistry, Biosynthesis, Metabolism, Distribution and Bioactivity in Plants and Animals—An Overview

**DOI:** 10.3390/ijms22189996

**Published:** 2021-09-16

**Authors:** Giuseppe Mannino, Carlo Pernici, Graziella Serio, Carla Gentile, Cinzia M. Bertea

**Affiliations:** 1 Department of Life Sciences and Systems Biology, Plant Physiology Unit, University of Turin, Via Quarello 15/A, 10135 Turin, Italy; giuseppe.mannino@unito.it (G.M.); carlo.pernici@edu.unito.it (C.P.); 2 Department of Biological, Chemical and Pharmaceutical Sciences and Technologies (STEBICEF), University of Palermo, Viale delle Scienze, 90128 Palermo, Italy; graziella.serio01@unipa.it

**Keywords:** indolamine, biostimulant, dietary supplements, cluster analysis, N-acetyl-5-methoxytriptamine

## Abstract

Melatonin is a ubiquitous indolamine, largely investigated for its key role in the regulation of several physiological processes in both animals and plants. In the last century, it was reported that this molecule may be produced in high concentrations by several species belonging to the plant kingdom and stored in specialized tissues. In this review, the main information related to the chemistry of melatonin and its metabolism has been summarized. Furthermore, the biosynthetic pathway characteristics of animal and plant cells have been compared, and the main differences between the two systems highlighted. Additionally, in order to investigate the distribution of this indolamine in the plant kingdom, distribution cluster analysis was performed using a database composed by 47 previously published articles reporting the content of melatonin in different plant families, species and tissues. Finally, the potential pharmacological and biostimulant benefits derived from the administration of exogenous melatonin on animals or plants via the intake of dietary supplements or the application of biostimulant formulation have been largely discussed.

## 1. Introduction

Melatonin (N-acetyl-5-methoxytriptamine) is an indolamine originally discovered in 1958 in extracts from bovine pineal gland, but this compound was first isolated and identified as a small molecule with a molecular weight of 232 Daltons in 1960 by Lerner [1]. The name was initially related to its ability to aggregate pigment granules (melanin) in the chromatophores of frog and fish skin. For more than 30 years, it was assumed that melatonin was exclusively produced in the pineal gland of animals, in which the indolamine acts as a neurohormone; however, nowadays it is known that melatonin is also produced by several organisms belonging the Eukarya and Bacteria domains, whereas no information has been found for Archea. Its extensive distribution has supported the theory that this indolamine is an ancient molecule retained throughout the evolution of all organisms [2,3]. Organisms such as *Rhodospirillum rubrum* [4,5], *Arthrospira platensis* (syn. *Spirulina platensis*) [6,7], *Lingulodinium polyedrum* (syn. *Gonyaulax polyedra*) [8,9,10] and *Pterygophora californica* [11] have acquired the ability to produce melatonin more than 2.5–3.5 billion years ago with the aim to mitigate the oxidative stress of reactive oxygen species (ROS) produced as a consequence of their aerobic metabolism [12]. Plants produce melatonin in different anatomical districts and in order to discriminate plant melatonin from melatonin produced by all other organisms, in 2004 the term ‘*phytomelatonin*’ was proposed [13].

In vertebrates, melatonin is rhythmically secreted by the pineal gland after photo-stimulation caused by dark or light-suppression [14] and it regulates the sleep–wake cycle and other seasonal rhythms. In these animals, the nocturnal melatonin peak also controls the reproductive capability [15]. The role of melatonin as circadian regulator appears to have evolved polyphyletically, since it was observed also in invertebrate animals, including the marine annelid *Platynereis dumerilii* (Polychaeta) [16,17]. Thanks to its antioxidant and radical scavenging properties [18], melatonin interacts also with the immune system of mammals acting as an immunostimulatory [19] and anti-inflammatory molecule [14]. On the contrary, despite some reports of a circadian melatonin production rhythm in both seaweed *Lingulodinium polyedrum* [20] and dicotyledon *Chenopodium rubrum* [21], it does not seem that melatonin may plays a role in the control of seaweed and plant photoperiodism [22]. It is more likely that melatonin might be involved in other plant functions, including growth and development, acting as an auxin-like molecule [23]. For instance, scientific evidence has suggested that melatonin could modify root architecture and morphogenesis [24,25,26], flowering processes [27], leaf senescence [28], and fruit ripening [29], chlorophyll, proline and carbohydrate content in leaves and fruits [30]. Recent findings also revealed its contribution as signalling molecule during biotic and abiotic stress [22,31,32,33,34], influencing plant defence responses against several pathogen attacks and enhancing stress tolerance to cold, drought, heavy metals, ultra violet radiations, or salt [22,35].

In this work, the chemistry and biosynthetic pathways involved in melatonin production in both animal and plant cells will be discussed. The two biosynthetic pathways will then be compared. Consequently, a meta-analytic approach will be employed in order to investigate the distribution of melatonin in the plant kingdom, highlighting the main sources of phytomelatonin. Finally, since melatonin has been shown to have important physiological roles in both plants and animals, the potential effects derived from the application of exogenous melatonin as a plant biostimulant or supplement for human use will be investigated.

## 2. Chemistry of Melatonin

**Figure 1:**
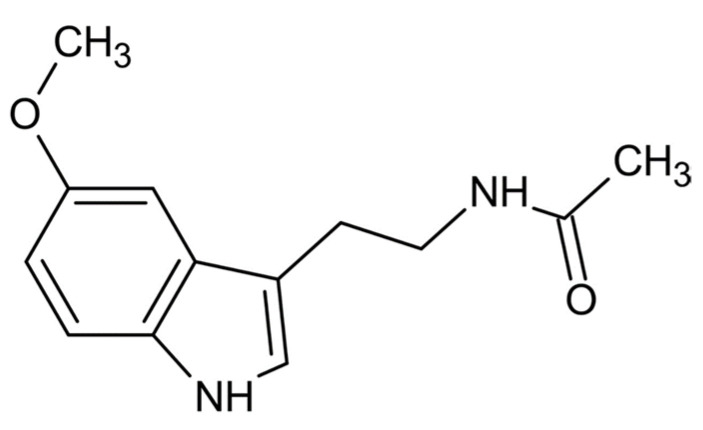
Chemical structure of melatonin.

From a physio-chemical point of view, pure melatonin resembles an off-white powder, having 232.28 g/mol as molecular weight and a density of 1.175 g/cm^3^. The melting point ranges between 116.5 °C and 118 °C; the boiling point is 512.8 °C [14]. From a chemical point of view, melatonin is identified by the chemical formula C_13_H_16_N_2_O_2_. The indole chemical scaffold is functionalized with a 3-amide group and a 5-alkoxygroup (Figure 1). Moreover, since it is originated starting from a molecule of tryptophan, is classified as an indolamine compound [36]. This particular chemical structure confers great stability by high resonance mesomerism.

**Figure 2:**
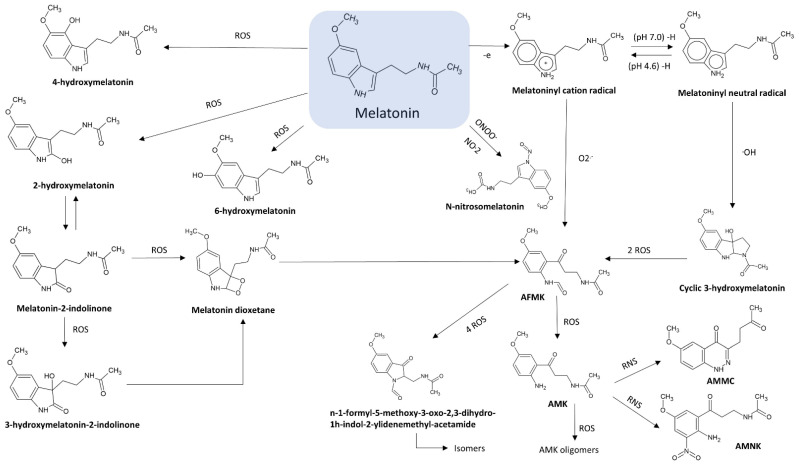
Melatonin metabolism and its related metabolites. ONOO^−^ = peroxynitrite; ROS = reactive oxygen species; RNS = reactive nitrogen species; O_2_^−^: superoxide anion; OH^−^: hydroxyl radical; AMFK: N1-acetyl-N2-formyl-5-methoxykynuramine; AMK: N1-acetyl-5-methoxykynuramine; AMMC: 3-acetamidomethyl-6-methoxycinnolinone; AMNK: N1-acetyl-5-methoxy-3 nitrokynura-mine.

Moreover, the 3-amide group and 5-alkoxygroup are also the main responsible of the amphiphilicity of this molecule. This property makes melatonin able to cross biological membranes and enter any cellular and subcellular compartments [36,37], allowing not only its easy distribution but also a high protection against oxidative stress in various cell compartments [36,38]. The antioxidant protection of melatonin is correlated both to its own redox active properties and to metabolites originated during its metabolism. Indeed, a series of new compounds having noteworthy antioxidant properties may be further generated by melatonin oxidation in a set of reactions known as melatonin antioxidant cascade [39,40]. Among these metabolites, cyclic 3-hydroxymelatonin (C3-OHM), N1-acetyl-N2-formyl-5-methoxykynuramine (AFMK), N1-acetyl-5-methoxykynuramine (AMK), 6-hydroxymelatonin (6-OHM), 2-hydroxymelatonin (2-OHM) are the most known (Figure 2).

### 2.1. N1-Acetyl-N2-Formyl-5-Methoxykynuramine (AFMK) 

Kynuramine compounds, such as AFMK and its de-formylated form (AMK), are molecules produced during tryptamine degradation. The redox activity and antioxidative properties of AFMK have been evaluated in several experimental models. Unlike antioxidants, such as vitamin C and vitamin E, AMFK can donate more than one electron [41]. In particular, Rosen and colleagues showed that AFMK can donate four electrons leading to the production of indolinone derivatives, such as Z-, E- isomers of N-(1-formyl-5-methoxy-3oxo-2,3-dihydro-1H-indol-2-ylidenemethyl)-acetamide and N-(1-formyl-2-hydroxy-5-methoxy-3-oxo-2,3-dihydro-1H-indol-2-ylmethyl)-acetamide [42]. However, AFMK was reported to be a less effective free radical scavenger than AMK and melatonin [43,44,45,46]. The antioxidant properties of AFMK were demonstrated also in biological models. In particular, Tan and colleagues showed that the addition of AFMK to calf thymus DNA in presence of a mixture of prooxidant agents strongly reduced in a dose-dependent way the levels of 8-OH-dG (an indicator of DNA damage) [41]. Moreover, in rat liver homogenates incubated with H_2_O_2_ and Fe^2+^, 100 µM AFMK inhibited lipid peroxidation (LPO) and improved cell viability, although Fe^2+^ chelation was not observed [41]. 

### 2.2. N1-Acetyl-5-Methoxykynuramine (AMK)

AFMK can be both enzymatically and non-enzymatically de-formylated resulting in the formation of AMK [47,48]. This compound showed a higher efficiency for scavenging ROS and preventing protein oxidation with respect to AFMK [49]. Radical scavenging action of AMK leads to the production of AMK oligomers, such as 3-acetamidomethyl-6-methoxycinnolinone and N1-acetyl-5-methoxy-3 nitrokynuramine. This property of AMK strongly depends on the environmental conditions [46]. Indeed, it has been observed that in aqueous solution, AMK is a radical scavenger stronger than melatonin, although it is a good scavenger also in nonpolar environment. In particular, AMK is a better OH^•−^ and NO scavenger than both melatonin [50] and AFMK [51]. 

### 2.3. 3-Hydroxymelatonin (C3-OHM)

Melatonin oxidation by reactive oxygen species (ROS) and reactive nitrogen species (RNS) scavenging may produce also C3-OHM. Experimental data showed an antioxidant protection by radicals. In particular, as with AFMK, also C3-OHM prevented DNA oxidation induced by Fenton reaction [52]. The presence of C3-OHM was always coupled to AFMK formation, both in vitro and in vivo experimentations [53]. The ratio between oxidants and melatonin affected the amount of melatonin oxidation products. In particular, higher were the ROS levels, more AFMK was produced [54]. Indeed, in this condition, C3-OHM can also be oxidised to AFMK. 

### 2.4. 6-Hydroxymelatonin (6-OHM)

6-Hydroxymelatonin (6-OHM), for the first time discovered in animal urine in the form of 6-hydroxymelatonin sulfate, is one of the major melatonin catabolites in animals. Experimental data showed that 6-OHM prevented lipid peroxidation [55] and DNA damage induced by environmental pollutants, chromium [56], and OH^•−^ generated by Fenton reaction [57]. Duan et al. also showed neuronal protection by 6-OHM in a model of ischemia/reperfusion-mediated injury. In this model, the anti-apoptotic action involved the inhibition of cytochrome C, inhibition of caspase 3 activity, and stabilization of the mitochondrial membrane potential [58]. Although the known protective effect of 6-OHM, a slight prooxidant activity was also shown. In particular, it was reported that 6-OHM caused oxidative DNA damage with double-strand breaks via redox cycling [59].

### 2.5. 2-Hydroxymelatonin (2-OHM)

Melatonin oxidation also leads to the production of 2-OHM, especially after scavenging of HClO [60], oxoferryl haemoglobin [61] and OH^•−^ [62]. Conversely to 3-OHM, 2-OHM is one the prevalent products of the hydroxylation of melatonin in plants. 2-OHM production is coupled to the formation of the keto tautomer melatonin 2-indolinone. In addition, in cytochrome C in vitro models the oxidation 2-OHM into AFMK was observed [63].

## 3. Biosynthesis of Melatonin

### 3.1. Biosynthetic Route in Plants

It has been shown that the cellular compartments with the highest melatonin levels in plants are mitochondria and chloroplasts [31]. This observation, together with the demonstrated localization of serotonin N-acetyltransferase (SNAT), one of the rate-limiting enzymes involved in melatonin biosynthesis, in chloroplasts [64,65] and in mitochondria [66], leads to hypothesize that these organelles are the major sites involved in the biosynthesis of this indolamine. The genes encoding for all the enzymes catalysing the whole melatonin biosynthetic pathway in plants have been discovered in several plant species, with the exception of one putative gene encoding for a tryptophan hydroxylase (TPH), which catalyses the conversion of tryptophan into 5-hydroxytryptophan [67]. In particular, this enzyme, already known in vertebrates, was only recently proposed in plants. 

**Figure 3:**
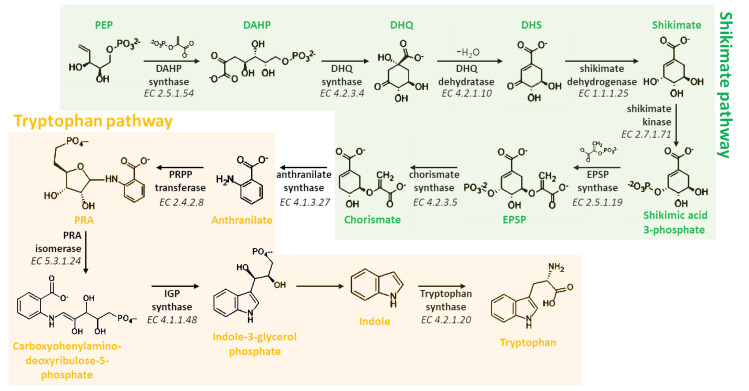
Biosynthetic pathway involved in the synthesis of tryptophan, the key compound for the formation of melatonin in plants. PEP: 2-phosphoenolpyruvate; DAHP: 3-deoxy-D-arabinoheptulosonate 7-phosphate; DHQ: 3-dehydroquinic acid; DHS: 3-dehydroshikimate; PEP: 2-phosphoenolpyruvate; EPSP: 5-enolpyruvylshikimate-3-phosphate; PRA: Phosporibosyl antranilate; PRAI: PRA isomerase; PRPP: phosphoribosylpyrophosphate; IGP: indole-3-glycerol phosphate; EC: enzyme commission number).

Melatonin biosynthesis begins with the amino acid tryptophan, a compound that plants are able to synthesize *de novo* via the shikimate pathway (Figure 3). This pathway consists of seven different steps that allow to the biosynthesis of all aromatic amino acids in plants, including tryptophan [68]. Briefly, 3-Deoxy-D-arabinoheptulosonate 7-phosphate (DAHP) synthase (EC 2.5.1.54) transforms phosphoenol pyruvate (PEP) and erythrose-4-phosphate in DAHP, that is then cyclized into 3-dehydroquinate (DHQ) by the action of the DHQ synthase (EC 4.2.3.4). Finally, shikimate is synthetized through the dehydration and dehydrogenation catalyzed by DHQ dehydratase (EC 4.2.1.10) and shikimate dehydrogenase (EC 1.1.1.25). Therefore, shikimate is phosphorylated by the shikimate kinase (EC 2.7.1.71) and converted in 5-enolpyruvylshikimate-3-phosphate (EPSP) by the EPSP synthase (EC 2.5.1.19). Finally, chorismate is formed through the activity of chorismate synthase (EC 4.2.3.5) that converts EPSP in chorismate, the essential intermediate in tryptophan biosynthesis (Figure 3). Chorismate is converted in anthranilate via anthranilate synthase (EC 4.1.3.27) that is consequently condensed with phosphoribosylpyrophosphate (PRPP), generating phosphoribosyl anthranilate (PRA). The ribose ring added in this last reaction is then opened by PRA isomerase (PRAI; EC 5.3.1.24), subjected to reductive decarboxylation in order to form indole-3-glycerol phosphate that is spontaneously converted into the indole scaffold. Finally, tryptophan is produced via the reaction of the indole with serine through the action of tryptophan synthase (TPS; EC 4.2.1.20) (Figure 3).

At least six enzymes are known to be involved in melatonin biosynthesis from tryptophan, indicating that multiple biosynthetic pathways may be present in this process. The six enzymes known to be involved in the synthesis of melatonin are: (i) L-tryptophan decarboxylase (TDC), (ii) tryptamine 5-hydroxylase (T5H), (iii) serotonin N-acetyltransferase (SNAT), (iv) acetylserotonin O-methyltransferase (ASMT), (v) caffeic acid 3-O-methyltransferase (COMT), and (vi) a putative tryptophan hydroxylase (TPH) not yet identified. 

**Figure 4:**
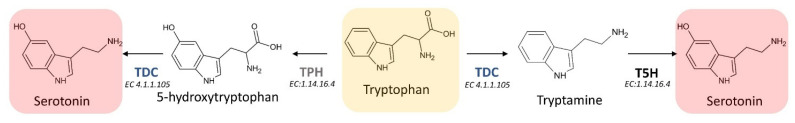
First two reactions of the melatonin biosynthetic pathway leading to the formation of the essential intermediate serotonin. TDC: L-tryptophan decarboxylase; TPH: tryptophan hydroxylase; T5H: tryptamine 5-hydroxylase; EC: enzyme commission number).

The first step of the melatonin biosynthetic process in plants is related to the production of serotonin from tryptophan. Two different pathways may be involved (Figure 4). The first way begins with the decarboxylation of tryptophan into tryptamine by TPH, and then tryptamine is hydroxylated to serotonin by TDC. On the other hand, another possibility first involves the hydroxylation of tryptophan into 5-hydroxytryptophan by TPH, and then the decarboxylation of 5-hydrotryptophan into serotonin by TDC. These routes are both possible, because TDC shows a good affinity for both tryptophan and 5-hydroxytriptophan in vitro [69]. However, it has been demonstrated that in plants is more frequent the decarboxylation than the hydroxylation as first step [69].

**Figure 5:**
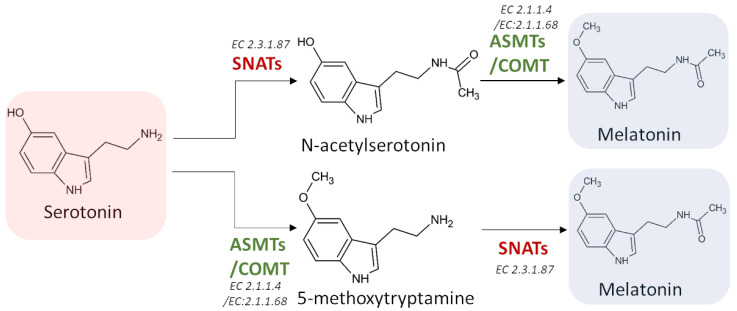
The last two potential reactions leading to the formation of melatonin. SNATs: serotonin N-acetyltransferase; ASMTs: acetylserotonin O-methyltransferase; COMT: caffeic acid 3-O-methyltransferase; EC: enzyme commission number.

Melatonin synthesis from serotonin is a two-step reaction involving three different enzymes (SNATs, ASMTs, and COMT) that may have various isoforms. The first enzyme catalyzes an acetylation, whereas the other two enzymes are methyltransferases [69] (Figure 5). As the tree enzymes exhibit a substrate affinity for serotonin, N-acetylserotonin, and 5-methoxytryptamine, also in this case the order by which the different enzymes act can vary [70,71,72]. 

**Figure 6:**
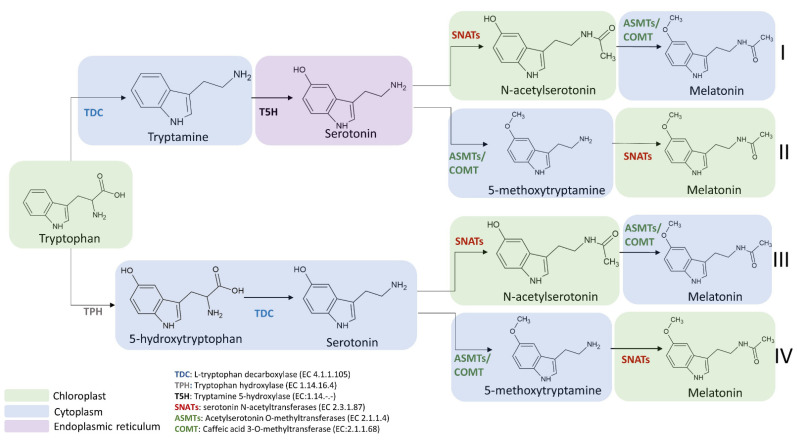
Subcellular localization of melatonin intermediates and enzymes involved in the transformation of tryptophan into melatonin in the different melatonin biosynthetic routes. TDC: L-tryptophan decarboxylase; TPH: tryptophan hydroxylase; T5H: tryptamine 5-hydroxylase; SNATs: serotonin N-acetyltransferase; ASMTs: acetylserotonin O-methyltransferase; COMT: caffeic acid 3-O-methyltransferase.

The enzymes involved in melatonin biosynthesis from tryptophan have a different distribution in plant cells. TDC is localized in the cytoplasm [73], T5H in the endoplasmic reticulum [39], SNAT is expressed in chloroplasts [39], whereas ASMT and COMT are in the cytoplasm [74]. Among the four possible melatonin biosynthetic pathways reported in Figure 6, the first and the second pathways result in serotonin synthesis in the endoplasmic reticulum, whereas the third and fourth in cytoplasmic environment [69]. ASMTs/COMT are exclusively located in the cytoplasm and SNATs in the chloroplast, the final subcellular sites of melatonin synthesis and accumulation may vary. For example, in the cytoplasm serotonin is rapidly metabolized into phenylpropanoid amides, such as feruloylserotonin, by the serotonin N-hydroxycinnamoyl transferase (SHT) [75] and melatonin is rapidly converted into cyclic 3-hydroxymelatonin (3-OHM) by the melatonin 3-hydroxylase (M3H), whereas in chloroplasts melatonin can be metabolized into 2-hydroxymelatonin (2-OHM) by the melatonin 2-hydroxylase (M2H) [76,77].

The selection of the pathway for melatonin biosynthesis depends on plant growth conditions. Indeed, under standard or stress conditions that do not cause a high accumulation of serotonin, the melatonin biosynthetic pathway proceeds from tryptophan via the intermediate tryptamine/serotonin/N-acetylserotonin up to melatonin [78] (Figure 6). In this pathway, serotonin levels are relatively low and this molecule is preferentially acetylated to N-acetylserotonin by SNAT due to its higher affinity (K_m_ = 0.385 mmol/L) for serotonin in comparison to ASMT (K_m_ = 1.035 mmol/L) and COMT (K_m_ = 3.396 mmol/L). The produced N-acetylserotonin is rapidly O-methylated into melatonin by either ASMT or COMT with a 30-fold higher catalytic efficiency than SNAT, leading to low levels of N-acetylserotonin. Based on previously published data, it was observed that COMT exhibits higher catalytic efficiency than ASMT at 37 °C but in vivo experiments, and it was observed that the activity of COMT to methylate N-acetylserotonin into melatonin was markedly inhibited due to the fact that COMT preferred methylate other substrates such as caffeic acid and 5-hydroxyconiferaldehyde [79,80]. These phenomena resulted in the functional loss of COMT activity for melatonin synthesis and a dominant role of ASMT in methylating N-acetylserotonin into melatonin (Figure 6).

On the other hand, during plant senescence or under abiotic stress conditions, plants tend to accumulate large amounts of melatonin intermediates (such as tryptophan, tryptamine and serotonin) [35]. Consequently, the biosynthetic route preferably proceeds from tryptophan via the intermediates tryptamine/serotonin/5-MT up to melatonin [69], where serotonin is O-methylated into 5-MT by COMT, and then it is acylated by SNAT leading to the formation of melatonin (Figure 6). However, it was observed that a serotonin boost was not proportionally correlated to a significant increase in melatonin level. Indeed, in several experimental conditions, despite the content of tryptophan and serotonin was slightly enhanced during the senescence, an equal increment of melatonin level was not observed. For example, data from senescent detached rice leaves have shown a difference of more than threefold in metabolic capacity between serotonin and melatonin synthesis [81,82]. This huge difference between the two compounds could be explained by the relatively low catalytic efficiencies of COMT and SNAT in senescence compared to those under normal growth conditions. However, despite SNATs auto-inhibition by serotonin was not currently observed and other regulatory roles of serotonin on the melatonin synthetic pathway are still unknown, low levels of melatonin and relatively high levels of 5-MT are obtained compared to N-acetylserotonin [83,84].

Despite the melatonin biosynthetic pathway under normal conditions producing more melatonin that in senescence and serotonin boost conditions, the melatonin levels are not related anyway to the levels of tryptophan and serotonin present in the cells. Consequently, the limiting step could be attributed to the production of N-acetylserotonin by SNAT [84]. Indeed, N-acetylserotonin must first cross the chloroplast membrane into the cytoplasm where ASMT or COMT can now transform it into melatonin [72,84].

Another potential route based on the studies conducted on T5H-deficient and T5H-suppression rice plants seems to be related to 5-hydroxytryptophan-mediated serotonin synthesis. In the investigated plants, the serotonin levels were much lower compared to control plants, but melatonin levels were higher. This interesting result was incompatible with the previously described melatonin biosynthetic pathways [85,86]. In this case, it was supposed that 5-HT is produced from tryptophan by the action of a putative TPH and then converted into serotonin by TDC. Given the low levels of serotonin and the high levels of melatonin found in T5H-deficient plants, the 5-HT pathway does not result in a serotonin boost but plays a key role in inducing melatonin levels.

Finally, other melatonin biosynthetic routes may exist [87,88], including those independent from the formation of serotonin. However, the involved enzymes are not yet identified and those already known seem not to be involved in this process [69].

### 3.2. Biosynthetic Route in Animals

A clear difference in melatonin synthesis between animals and plants concerns the availability of the precursor tryptophan. Unlike plants, animals cannot synthetize tryptophan *de novo* (Figure 3), but it must be taken in through the diet. Similarly to plants, also animal mitochondria are the main biosynthetic sites and the compartments with the highest concentration of melatonin [89]. The melatonin biosynthetic pathway in mammals was first discovered by Axelrod’s group in 1960, and now it is well defined [90]. However, the classic melatonin biosynthetic pathway has been expanded in all vertebrates and can be applied also to other animals, including insects [91].

**Figure 7:**
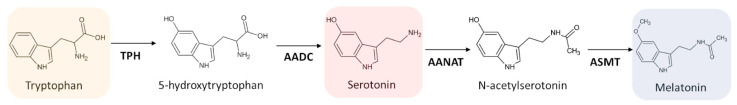
The classic melatonin synthetic pathway in animals. TPH: tryptophan hydroxylase; AADC: aromatic amino acid decarboxylase; AANAT: aralkylamine N-acetyltransferase; ASMTs: acetylserotonin O-methyltransferase.

The pathway involves five enzymatic steps (Figure 7). In the first step, tryptophan is hydroxylated to 5-hydroxytryptophan by TPH, that is subsequently decarboxylated to serotonin (5-hydroxytryptamine) by the aromatic amino acid decarboxylase (AADC). The two final steps were cryptic for several years. Indeed, it was not clear neither the biosynthetic location of melatonin nor the enzymes involved in the synthesis. In 1960, when the melatonin biosynthetic pathway was discovered in animals, it was proposed that serotonin is acetylated by AANAT to N-acetylserotonin exclusively in pineal gland and liver [92]. However, since ASMT was originally detected in the pineal gland, it was wrongly suggested that melatonin production was not localized in the liver. For this reason, melatonin was initially classified as a pineal-related neurohormone [93]. However, to date it is well known that melatonin is also produced by many peripheral tissues and organs, such as retina, Harderian gland, ovary, testis, bone marrow, lymphocytes, hepatic cholangiocytes, gut, and skin [93]. In particular, it was observed that skin and gut produce more melatonin than the pineal gland [94]. Thus, the concept of melatonin as a neurohormone was modified and the observed ubiquitous presence of extra-pineal melatonin in mammals was explained [95,96].

Later, it was observed that the enzyme activity of ASMT for N-acetylserotonin was approximately 14-fold higher in presence of serotonin, thus it was supposed that N-acetylserotonin was the preferable substrate of ASMT rather than serotonin [97]. Based on these observations, it was assumed that serotonin is first acetylated to form N-acetylserotonin by AANAT and the resulting N-acetylserotonin methylated to melatonin by ASMT (Figure 7). AANAT is widely accepted as the limiting factor for the production of melatonin. Indeed, in mammals the main melatonin biosynthetic regulatory factor is light, particularly blue light (~420–480 nm) [98]. In order to achieve a relatively long-term effect [95], this kind of irradiation during the day immediately suppresses melatonin biosynthesis by inhibiting the activity of AANAT both via protein dephosphorylation and gene down-regulation [99]. Other factors that may impact on animal melatonin biosynthesis include food intake, temperature alterations, and diseases [100].

### 3.3. Focus on the Enzymes Involved in the Biosynthetic Routes

**Figure 8:**
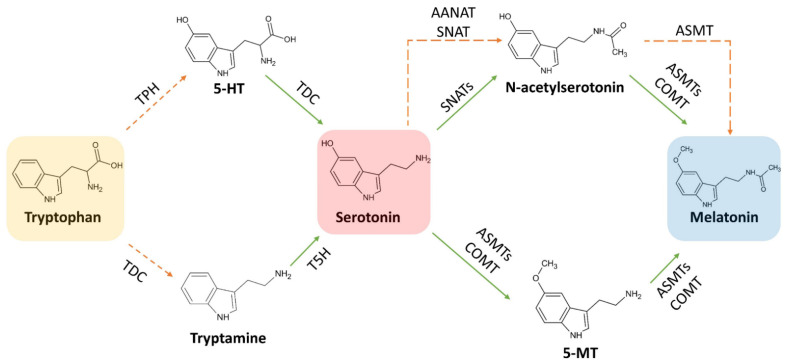
The melatonin biosynthetic pathway in mitochondria (orange broken arrows) and in chloroplasts (solid green arrows). In plants both pathways are probably present: when the main chloroplast pathway is interrupted (like in Sekiguchi mutant rice) the mitochondrial pathway takes over to compensate for the lack [85,86]. TDC: L-tryptophan decarboxylase; TPH: tryptophan hydroxylase; T5H: tryptamine 5-hydroxylase; SNATs: serotonin N-acetyltransferase; ASMTs: acetylserotonin O-methyltransferase; COMT: caffeic acid 3-O-methyltransferase.

As previously described, the biosynthetic pathways involved in the synthesis of melatonin in both plants and animals have some similarities and differences, mainly related to the enzymes, their substrate affinity and cellular localization. Moreover, differently to animals, plants have an additional melatonin biosynthetic pathway that proceeds from serotonin to 5-MT with the consequent acetylation of this intermediate to melatonin (Figure 8). This evidence is based on some recent studies in which it was reported how the two melatonin-generating sites can crosstalk to maintain a stable supply of melatonin in plants when the chloroplast pathway is blocked. In this particular context, plants can switch the chloroplast-based production into mitochondria, using a biosynthetic pathway similar to that found in animals. However, some differences related to the origin and activity of enzymes have been found. In this section, the main differences in the activity observed for plant and animal enzymes involved in melatonin biosynthesis will be investigated and discussed.

#### 3.3.1. L-Tryptophan Decarboxylase (TDC) (EC 4.1.1.105)

TDC is the enzyme catalyzing the conversion of 5-hidroxytryptophan into serotonin [73]. It was originally identified in *Catharanthus roseus* as a soluble cytosolic and homodimeric protein, composed by monomers having molecular weight equal to 54,000 u.m.a. TDC of *C. roseus* showed higher substrate affinity to tryptophan (K_m_ = 0.075 mmol/L) compared to 5-hydroxytryptophan (K_m_ = 1.3 mmol/L). On the contrary, TDC could not accept L-DOPA (dioxyphenylalanine) or phenylalanine as substrates [101]. TDC proteins purified from *Ophiorrhiza pumila*, *Oryza sativa* and *Rauvolfia verticillata* showed K_m_ values for tryptophan (0.72, 0.69 and 2.89 mmol/L, respectively), 10-fold higher than the values showed for TDC purified from *C. roseus.* However, it is also known that other species, including *Oryza sativa*, expressed multiple TDC proteins in contrast to *C. roseus* that has only one isoform [102,103].

#### 3.3.2. Tryptamine 5-Hydroxylase (T5H) (EC 1.14.-.-)

**Figure 9:**
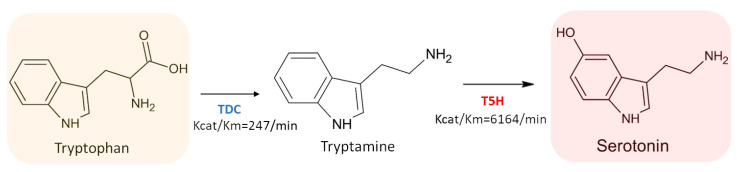
Synthetic capacity of serotonin from tryptophan.

Regarding the hydroxylation reaction occurring during melatonin biosynthesis, it is predominantly mediated in plants by two cytochrome P450-dependent monooxygenases (T5H and the putative TPH). Likely aromatic amino acid hydroxylase, both require tetrahydrobiopterin as a co-substrate [104]. Tryptamine 5-hydroxylase (T5H) belongs to the cytochrome P450 monooxygenase family [105], and it is responsible for the addition of one hydroxyl group to the 5 position of tryptamine. This reaction leads to the formation of 5-hydroxytryptamine [67]. T5H displays not only a high substrate affinity for tryptamine (K_m_ = 0.0073 mmol/L), but also a high turnover number (K_cat_ = 45/min) [105]. However, T5H enzyme does not catalyze the conversion of tryptophan into 5-hydroxytryptophan [105]. The catalytic efficiency (K_cat_/K_m_) measured for rice T5H was 6164/min. This value was 25-fold higher than the value measured for rice TDC (K_cat_/K_m_ = 247/min) suggesting a rapid conversion of tryptamine produced by TDC to serotonin (Figure 9). This data could explain the high content of melatonin in close-related species [106,107]. A suggestion of the existence of one or more enzymes involved in the synthesis of melatonin comes from Sekiguchi mutant rice, which completely lacks in T5H activity, but nevertheless it is able to synthetize melatonin at lower levels compared to the wild type [86]. Further analysis showed that Sekiguchi rice switched the melatonin synthetic pathway from the classic plant type using a route similar to that used by animals, in which tryptophan is hydroxylated to 5-hydroxytryptophan by a tryptophan hydroxylase (TPH). On the other hand, although no animal TPH homologs were detected in plant genome [85], other scientific evidences support the presence of TPH-like enzymes in plants. However, this route exhibits a very low serotonin biosynthetic flux rate if compared to the main melatonin biosynthetic plant pathway. For example, (i) *Griffonia simplicifolia* seeds are notoriously rich in 5-hydroxytryptophan, with amounts justifiable by assuming the presence of TPH-like enzymes [67]; (ii) in in vitro cultures of St John’s wort (*Hypericum perforatum* L.) was observed that the synthesis of serotonin mainly occurs via 5-hydroxytryptophan when 14C-tryptophan was added in culture medium, suggesting that it was produced from tryptophan by a TPH-like enzyme [108]; (iii) the soluble fraction of rice root extracts exhibited a tetrahydropterin-dependent amino acid hydroxylase activity, similar to TPH [109]; (iv) Park and colleagues observed a concomitant increase of the content of melatonin, tryptamine, tryptophan and 5-HT in transgenic rice plants (cv. Dongjin) with transcriptionally suppression of T5H with respect to the wild type [85].

#### 3.3.3. Serotonin N-Acetyltransferase (SNAT) (EC 2.3.1.87)

SNAT, also known in animals with the name of aralkylamine N-acetyltransferase (AANAT) [110], catalyzes the transfer of the acetyl group of Acetyl-CoA to the primary amine of serotonin, producing CoA and N-acetylserotonin. The comparison of plant SNATs and animal AANAT revealed clear differences in enzyme kinetics. Concerning the catalytic activity (V_max_/K_m_), sheep AANAT activity is 10-fold higher than rice SNAT. Moreover, it was shown that SNATs can accept various amine substrates, including serotonin, tryptamine, and 5-MT, with different affinity. In particular, the preferred substrate of AANAT is serotonin, whereas for SNAT is generally 5-MT. In rice SNAT has comparable affinity for serotonin and 5-MT (K_m_ = 0.385 mmol/L, 0.375mmol/L), while in *A. thaliana* SNAT affinity for 5-MT is sixfold higher than that of serotonin (K_m_ = 0.051 mmol/L, 0.309 mmol/L) [70]. Moreover, considering the turnover number of SNAT in *A. thaliana*, the catalytic efficiency (K_cat_/K_m_) for the conversion of 5-MT to serotonin is 23-fold higher than that recorded for the conversion of serotonin into melatonin. The genes encoding for SNATs have been isolated and identified in many vertebrates, yeasts and bacteria. Recently, they were also found in different algae and plants, including *Chlamydomonas reinhardtii* [111], *Pyropia yezoensis* [112], *Oryza sativa* [113], *Arabidopsis thaliana* [84] and *Pinus taeda* [114]. In plants, SNATs were mainly identified in chloroplast [84,111,113]. On the other hand, the enzyme AANAT from vertebrates shows homology with an alphaproteobacterial enzyme and it is inherited via mitochondria [115]. Based on the theory of endosymbiosis proposed by Sagan, α-proteobacteria are the precursors of mitochondria, while cyanobacteria of chloroplasts [116]. This theory led to the hypothesis that these organelles inherited the melatonin synthetic machinery from their prokaryotic ancestors.

Despite the similar enzymatic activity of SNAT and AANAT, the major difference is related to the stabilization mechanism of these enzymes. Indeed, AANAT contains regulatory flanking regions which have not been identified in plant SNATs. In several mammals, especially primates and ungulates, the stabilization of AANAT prevails over transcriptional up-regulation of the gene and it is decisive for post-transcriptional regulation of circadian AANAT rhythmicity [117].

#### 3.3.4. Acetylserotonin O-Methyltransferase (ASMT) (EC 2.1.1.4)

ASMT is an enzyme catalyzing the final reaction in melatonin biosynthesis, converting N-acetylserotonin to melatonin. It is also known as hydroxyindole-O-methyltransferase (HIOMT) [118]. Plant ASMTs lack in homology with the animal isoforms, causing the impossibility to identify and clone them until 2011 [119]. In the same year, the gene sequence of the first ASMT was identified in rice, and the protein was finally purified. The purified recombinant rice ASMT displayed low enzyme activities for N-acetylserotonin at 30 °C (K_m_ = 0.864 mmol/L; V_max_ = 0.21 pkat/mg protein) [120], showing instead 2800-fold higher catalytic efficiency at the optimal temperature of 55 °C (K_m_ = 0.222 mmol/L; V_max_ = 150 pkat/mg protein) [121]. Being TDC and SNAT slightly tolerant to high temperatures [121], it was suggested that melatonin synthesis is positively affected by high temperature in plants [122]. However, because the highest K_m_ value towards serotonin and the lowest value towards N-acetylserotonin, ASMTs use serotonin as a substrate only when there are high levels of serotonin in cellular environment. [83]. After the isolation of the first ASMT isoform from rice, in the following years other genes encoding for similar proteins were identified with a homology of about 50% in both monocotyledonous (oat, wheat, and barley) and dicotyledonous (grape, coffee, castor bean, alfalfa and poplar) plant species. However, due to the low homology with rice ASMT gene, at the beginning it was assumed that the ASMT-like genes discovered in dicotyledonous species encoded for proteins with no ASMT-like activity [115]. Nevertheless, Schröder and colleagues isolated an ASMT isoform from apple that had an amino acid sequence 39.7% similar to the rice ASMT protein, but that exhibited ASMT activity similar to the rice enzyme. This finding allowed to suppose that the same mechanism could occur for ASMTs with higher sequence homology to other plant species [83]. A similar case was also reported for ASMT gene present in *Arabidopsis thaliana*. Despite the fact that the gene sequence showed only 31% of identity to the rice ASMT gene, it showed an higher ASMT activity (0.11 pkat/mg protein) [84].

#### 3.3.5. Caffeic Acid 3-O-Methyltransferase (COMT) (EC 2.1.1.68)

It is well known that COMT plays a pivotal role in the lignin biosynthetic pathway [123] and in the methylation of several substrates, including caffeic acid, 5-hydroxyconiferaldehyde, and quercetin [124]. However, it also covers an important role in melatonin synthesis by methylating N-acetylserotonin [125]. Like ASMTs, also COMT can O-methylate both N-acetylserotonin and serotonin [72]. Concerning kinetic parameters, it was recorded that in Arabidopsis COMT showed higher affinity (K_m_ 0.233 mmol/L) and V_max_ (30 pkat/mg protein) towards N-acetylserotonin than ASMT (K_m_ = 0.456 mmol/L; V_max_ = 0.11 pkat/mg protein), resulting in a 636-fold higher catalytic efficiency of COMT compared to ASMT. Arabidopsis COMT also converts serotonin into 5-methoxytryptamine, with much lower affinity (3.396 mmol/Land) and V_max_ (8.8 pkat/mg protein) than that associated with conversion of N-acetylserotonin into melatonin [84]. Similar values were also measured for rice COMT [79]. Finally, in *comt* knockout mutants of *Arabidopsis* [72] and COMT suppressed rice lines [79], a marked reduction in the synthesis of melatonin was observed, assuming an essential role of this enzyme in melatonin biosynthetic pathway.

## 4. Distribution of Melatonin in Plants

Several plant species are able to produce large amount of melatonin, which is consequently stored in specialized tissues or organs. Melatonin levels in plants vary from undetectable to very high concentrations [126]. Indeed, in some cases the levels of melatonin are comparable to that of animals, with values ranging from few pg to ng per g of fresh weight (FW). However, it has previously reported that some plants can produce higher amounts of melatonin in comparison to animals. Several authors assumed that plants grown under unfavourable conditions, including heat or cold stress, exposure to soil pollutants, or bacterial infection, have the capacity to up-regulate phytomelatonin production [127]. Furthermore, since phototrophic organisms need an higher antioxidant environment to protect the appropriate functioning of photosystems, the very high concentration of phytomelatonin in chloroplasts should not be surprising [128]. The stress promoting melatonin production was also observed in animals, suggesting the key role of melatonin to make organisms more resistant to stress conditions.

In this work, a database consisting of published articles in which the phytochemical composition of different plant raw materials containing melatonin was built. The database originally contained 2485 entries, which were individually analyzed to select papers that provided an accurate melatonin content via HPLC methodology (*n* = 47) [38,115,129,130,131,132,133,134,135,136,137,138,139,140,141,142,143,144,145,146,147,148,149,150,151,152,153,154,155,156,157,158,159,160,161,162,163,164,165,166,167,168,169,170,171,172,173]. Furthermore, information regarding the species binomial name, plant family, common name, and plant part used for the extraction was extrapolated along with the melatonin content. Data reported using different measurement units were homogeneous and fixed for the water content when necessary. Consequently, all data were expressed as ng melatonin per gram of FW. The total number of selected species was 131, and the estimated 493 average melatonin content was 579.38 ± 1513.28 ng per g of FW.

### 4.1. Phytomelatonin Content within Plant Species

**Figure 10:**
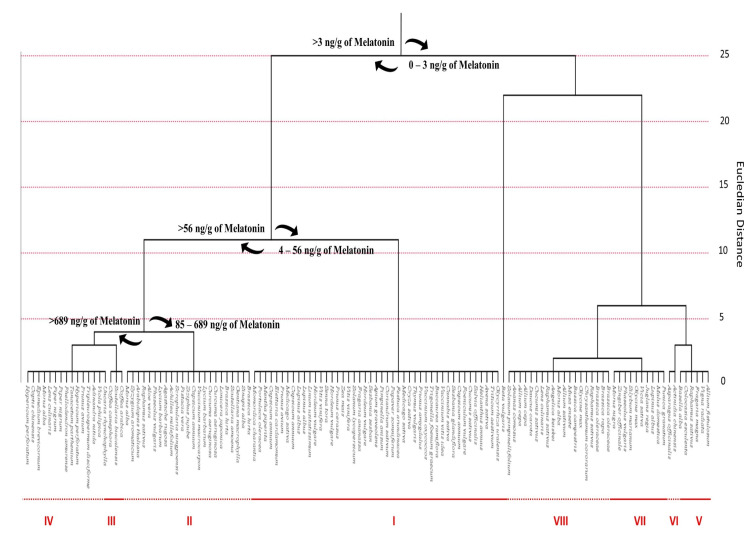
Cluster distribution of melatonin within plant species, according to previously published data [38,115,129,130,131,132,133,134,135,136,137,138,139,140,141,142,143,144,145,146,147,148,149,150,151,152,153,154,155,156,157,158,159,160,161,162,163,164,165,166,167,168,169,170,171,172,173]. Data are expressed as ng melatonin per g of FW Euclidean distances, calculated with centroid method. Statistical analysis and graphical representation were made using SPSS v. 24 software.

In order to underline the statistical linkage between different plant species, a cluster analysis was performed by considering as category the plant species and as variable the melatonin content (ng/g) (Figure 10). Squared Euclidean distances were calculated by using the centroid method. The estimated average melatonin content was 579.38 ± 1513.28 ng per g of FW. These data confirm the considerable variability of the melatonin content in plant species. Generally, aromatic and medicinal plants show significantly higher levels of phytomelatonin than fleshy fruits and seeds, with coffee seeds as the only exception. In particular, among the 131 analyzed species, *Coffea* genus had the highest phytomelatonin content, with values ranging from 5800 ng/g to 7466.667 ng/g. Additionally, medicinal and officinal plants, such as mint (*Mentha piperita*), sage (*Salvia officinalis*), thyme (*Thymus vulgaris*), St. John’s wort plant (*Hypericum perforatum*), barrenwort (*Epimedium brevicornum*), Amur cork tree (*Phellodendron amurense)*, Chinese goldthread (*Coptis chinensis*), *Scutellaria baicalensis*, *Adinandra nitida* and *Tripleurospermum disciforme* revealed an interesting content in phytomelatonin. Among common fruits, tomato (*Solanum lycopersicum)*, goji berry (*Lycium barbarum),* sweet and sour cherry fruits (*Prunus avium* and *Prunus cerasus)* and cranberry (*Vaccinium macrocarpon*) were the most representative with an average phytomelatonin content of 9.44 ng/g, 530 ng/g, 15.050 ng/g and 11.370 ng/g, 96 ng/g, respectively. Concerning cereals, rice (*Oryza sativa*) has the highest phytomelatonin content (55.55 ng/g) and generally all the analyzed species among the Poaceae family show high phytomelatonin levels. In particular, oat (*Avena sativa)* and common wheat (*Triticum aestivum*) have very high phytomelatonin content (31.533 ng/g and 33.425 ng/g, respectively), while lower content are recorded for barley (*Hordeum vulgare*) and corn (*Zea mays*) (12.127 ng/g and 11.15 ng/g, respectively). Statistical analysis evidenced eight different clusters according to the melatonin content. The cluster I contained the largest number (*n* > 40) of plant species. These plants have a melatonin content in different organs (flowers, seeds, leaves, roots, and fruits) ranging from 4 ng to 56 ng per g of FW. The other seven clusters largely vary in the melatonin content.

The cluster III is represented by only three species having a content of melatonin higher than 5800 ng/g (Figure 10). On the other hand, the clusters V–VIII included plant species with a very low melatonin content, ranging from 0 to 3 ng per g of tissue. Plants included in these clusters counted melatonin in different organs or tissues, such as flowers, seeds, bulbs, sprouts, hypocotyls, and roots. This finding suggests that the accumulation site of melatonin in plants may vary according to plant species.

### 4.2. Phytomelatonin Content within Plant Families

**Figure 11:**
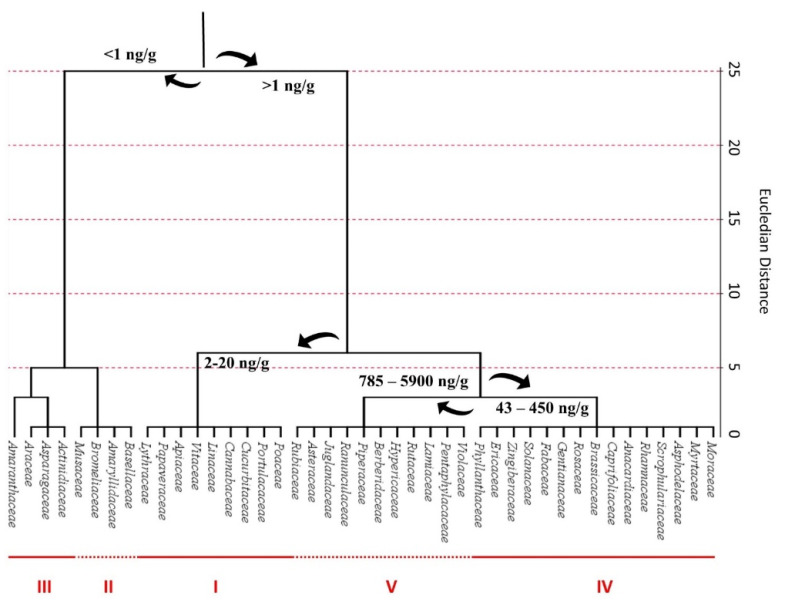
Cluster distribution of melatonin within plant families, according to previously published data [38,115,129,130,131,132,133,134,135,136,137,138,139,140,141,142,143,144,145,146,147,148,149,150,151,152,153,154,155,156,157,158,159,160,161,162,163,164,165,166,167,168,169,170,171,172,173]. Euclidean distances were calculated with centroid method. Data were expressed as ng melatonin per g of FW. Statistical analysis and graphical representation were made using SPSS v. 24 software.

After a systematic grouping of the identified plants according to their family, cluster analysis revealed that the 131 analyzed species belonged to 35 different families (Figure 11). The highest phytomelatonin content was reported for the Rubiaceae family, with an average phytomelatonin content of 5885 ng/g. Rubiaceae family includes the *Coffea* species, whose seeds notoriously have the highest phytomelatonin content. The Rubiaceae phytomelatonin content was twofold higher compared to species belonging to Violaceae, Pentaphylacaceae and Juglandaceae families. Other families noteworthy for their high phytomelatonin content were Lamiaceae, Rutaceae, Hypericaceae, Berberidaceae, Piperaceae, Ranunculaceae and Asteraceae.

The results of the cluster analysis showed the presence of five different clusters with an outlier value. The cluster I was composed by nine families with a melatonin content ranging between 2 and 20 ng/g. The clusters II and III contained four and three families respectively, with a melatonin content lower than 1 ng/g. Among them the most representative were Basellaceae, Amaryllidaceae, Bromeliaceae, Musaceae, Actinidiaceae, Asparagaceae and Araceae; the families that included species with the highest melatonin content were included in the cluster V. Finally, the cluster IV contained fifteen families with a melatonin content ranging from 43 to 450 ng/g and a punctual outlier (Amaranthaceae family) that had a very low melatonin content.

### 4.3. Phytomelatonin Content in Plant Tissues

**Figure 12:**
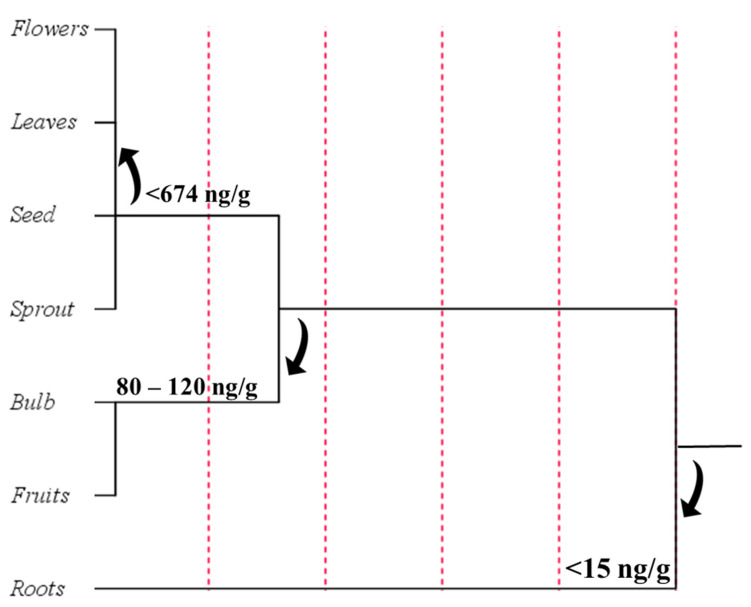
Cluster distribution of melatonin in plant organs, according to previously published data [38,115,129,130,131,132,133,134,135,136,137,138,139,140,141,142,143,144,145,146,147,148,149,150,151,152,153,154,155,156,157,158,159,160,161,162,163,164,165,166,167,168,169,170,171,172,173]. Euclidean distances were calculated with centroid method. Statistical analysis and graphical representation were made using SPSS v. 24 software.

In order to understand which plant organ was designated to preferentially accumulate phytomelatonin, a further cluster analysis was performed after grouping the plant raw materials into seven categories: (i) flowers; (ii) leaves; (iii) seeds; (iv) sprouts; (v) bulbs; (vi) fruits; (vii) roots (Figure 12).

Cluster analysis revealed that the plant organs with the highest melatonin content were flowers, with an average content equal to 694.08 ng/g, immediately followed by leaves and seeds that recorded a similar phytomelatonin value but a larger standard deviation. Cluster analysis showed that the fruits contained low phytomelatonin content (89.05 ng/g) with respect to other tissues. The tendency of plants to mainly store melatonin in flowers and leaves could be explained by the antioxidant and radical scavenger properties of this compound that normally helps the more exposed organs to counteract potential stressful conditions. These data could be explained by the constitutive presence of other antioxidants in the fruits that effectively protect pigments from oxidative menaces. On the other hand, the low value recorded for phytomelatonin content in fruits may be due to influence of harvesting time and post-harvest condition, in addition to ripeness stage of fruits, along with the variability of the different cultivation conditions and environmental factors that normally may affect fruit maturation [127].

## 5. Role of Phytomelatonin in Plants

Melatonin is a plant endogenous compound with a wide variety of functions, ranging from hormonal to antioxidant. Recently, part of the functions and action mechanisms of this indolamine has been clarified, and this compound is now considered a plant master regulator and a valuable response marker to different kind of stresses. For example, in lupin and tomato, it was observed that melatonin content may simply increase by cultivating plants in field or chambers [127,174]. A similar trend has been also observed in water hyacinth plants [175], grape berry skin [170], and cherry fruits [162]. These phenomena may be related to the adverse environmental factors that are certainly more frequent in field than in greenhouse. Indeed, it has been reported that various environmental factors, such as temperature oscillations, light–dark cycles, UV radiation, and water availability, may strongly influence melatonin production [176]. In rice plants exposed to the high temperature (55 °C) and/or to dark conditions higher melatonin levels have been measured thanks to the modulation of the enzymatic activity of SNAT and HIOMT [122]. Water restrictions during the growth of lupin seedlings caused an increase in endogenous melatonin, which was fourfold higher than that observed in well-irrigated plants [174]. Melatonin has also been demonstrated to positively influence the cold resistance of plants, seeds, callus, cultured cells, and shoot explants, with a promising role for the long-term storage of germplasm for plant cell culture and for his protective role against chilling stress [177].

Given the predominant role of melatonin in counteracting abiotic stress phenomena, the potential to increase the biosynthetic capacity of this indolamine in plants was investigated. Some scientific evidences showed that melatonin-rich transgenic rice plants were able to grow more robust and with a greater height and biomass than wild type plants under field conditions [172]. Similar effects were also demonstrated for melatonin-rich transgenic woodland tobacco (*Nicotiana sylvestris*) plants, that showed improved resistance to UV-B radiation at DNA level with respect to wild type plants [178]. Moreover, transgenic rice seedlings expressing human SNAT showed to have greater cold resistance compared to wild type [179], indicating once again the protective role of melatonin against cold and abiotic stress in general. However, the problem related to plant engineering is due to the fact that this practice is not well received by many countries, and therefore not permitted. Consequently, in recent decades the hypothesis of melatonin application has been proposed, in order to evaluate if this indolamine applied exogenously could show similar effects to those measured in melatonin-rich transgenic plants. Moreover, the exogenous application of melatonin to plants may be considered a sustainable agronomic practice, belonging to the biostimulation process. Indeed, the European Biostimulants Industry Council (EBIC) defined biostimulants as “*Substances and/or microorganisms whose function when applied to plants or the rhizosphere is to stimulate natural processes to benefit nutrient uptake, nutrient use efficiency, tolerance to abiotic stress, and/or crop quality, independently of its nutrient content*” [180,181]. Therefore, the exogenous treatment of plants with melatonin perfectly fits with this description. Finally, since melatonin is normally and physiologically produced by both plants and humans and does not exert an evident toxicity even at high dosages [182], it should be considered also as a safe chemical.

In the following sections, the potential effects derived from the exogenous application of melatonin at the morphological, physiological, biochemical, and molecular level will be discussed.

### 5.1. Melatonin as a Promoter or Inhibitor of Plant Growth

The treatment of plants or seeds with exogenous melatonin revealed both a promoting and inhibiting effect on shoot, leaf, root growth and yield of several species in a concentration-dependent manner. Studies on *Lupinus albus* showed that the application of melatonin may act as a vegetative growth promoter in cotyledons [183] and etiolated lupin [184], by inducing growth of hypocotyls after the treatment at micromolar concentrations. On the other hand, this molecule seems to have an inhibitory effect at higher concentrations, displaying an auxin-like effect [185]. In particular, the growth-promoting effect of melatonin turned out to be 63% similar to that of indole-3-acetic acid (IAA) [184]. This auxin-like effect was observed by several authors on different plant species. For example, the growth promoting activity of melatonin at nanomolar concentrations was evaluated on oat (*Avena sativa*), wheat (*Triticum aestivum*), barley (*Hordeum vulgare*), and canary grass (*Phalaris canariensis*). In these studies, it was calculated that melatonin activity ranged from 10% (oat coleoptiles) to 55% (barley coleoptiles) with respect to IAA [132]. In the same study, a concentration-dependent growth-inhibitory effect at micromolar concentration was also observed, especially on roots (56%-86% with respect to IAA) in canary grass and to 86% in wheat roots [132]. Similar effects were also described in red cabbage (*Brassica oleracea rubrum*) and in mustard (*Brassica juncea*) roots, where the treatment with 0.1 μM melatonin showed a stimulatory effect on root growth, whereas an inhibitory response was observed after treatment with 100 μM [185,186].

The role of melatonin in the induction of rhizogenesis was initially demonstrated in 2007 by Marino and colleagues, who observed a root primordia generation from lupin pericycle cells after melatonin treatment. This rhizogenic effect was confirmed in the following years by different authors and in a number of plants, including cucumber (*Cucumis sativus*) [187], in four cherry rootstocks (*Prunus cerasus; P. cerasus* × *P. canescens; P. avium* × *P. mahaleb; P. avium* × *P. cerasus*) [26], in pomegranate (*Punica granatum*) [188], in *Arabidopsis thaliana* [189,190], and in rice (*Oryza sativa*) [191]. Moreover, studies conducted on transgenic rice seedlings overexpressing sheep serotonin N-acetyltransferase, showed that the melatonin levels were 10-fold higher with respect to the wild type seedlings, and had a twofold enhancement of the root growth, demonstrating a direct relationship between the endogenous melatonin level and the root growth rate [191]. Consequently, due to some similarities between melatonin and IAA, such as the apparent auxin-mimetic action exerted by melatonin, the common precursor and the structural analogy, several authors mistakenly identified the melatonin as an auxin-mimetic molecule able to promote vegetative growth [25]. However, nowadays it is known that melatonin does not stimulate IAA biosynthesis or mimic its actions, but it affects the root growth in an auxin-independent manner [189,190]. This evidence comes from a study in which the expression of an auxin-inducible marker (DR5::GUS) was evaluated in roots of *Arabidopsis thaliana* after the treatment with melatonin or two auxins (IAA and NAA). In this work, the authors observed that GUS was exclusively expressed after IAA and NAA treatments [190]. Another observation comes from Zhang and colleagues, who not only compared the morphological root architecture of plants treated with melatonin to untreated ones, but also performed a whole-transcriptome sequencing analysis (RNA-Seq) [192]. In their experimental conditions, the authors observed that 121 genes were up-regulated and 196 genes were significantly down-regulated in melatonin-treated roots [192]. They also observed an increased number of lateral roots in treated plants compared to untreated. Moreover, because the auxin-related genes exhibited minimal expression differences, they confirmed the hypothesis that melatonin affects the root pattern in an auxin-independent manner [189,190].

However, melatonin treatment seems to affect endogenous auxin levels in many plants [193,194]. For example, exogenous melatonin pre-treatment increased IAA and Indole-3-butyric acid (IBA) of tomato plants and mustard seedlings [185,195], whereas 600 μM melatonin decreased endogenous IAA level in Arabidopsis, repressing auxin synthesis related genes (*YUC1, YUC2, YUC5, YUC6, TAA1, TAR2*) and IAA efflux components (*PIN1, PIN3, PIN7*), then remarkably suppressed primary root growth [196]. Moreover, melatonin pre-treatment at relatively low dosage (50 µM) activated the expression of auxin efflux genes (*PIN1, PIN3* and *PIN7*) and signalling transduction genes (*IAA19* and *IAA24*) to promote adventitious root formation in Arabidopsis, tomato, and rice plants [195,197,198]. Further transcriptome analysis on Arabidopsis showed that sixteen IAA pathway-related genes were modulated upon melatonin treatment [197], and an improved root growth by low (10 and 50 µM) concentration of melatonin [199] was observed, however these positive effects were absent in several mutants of auxin transporters [197]. In rice, 10–20 µM melatonin pre-treatment increased the expression levels of *OsIAA1*, *OsIAA9*, *OsIAA10*, *OsIAA20*, and *OsIAA27* [198]. These results suggested that melatonin might partially function as an IAA mimic molecule at low concentration, but the question remains still open.

### 5.2. Melatonin Affects Seed Germination and Plant Performances

Seed priming is an effective method to improve sowing material and consequently enhance seed viability and future plant strength. This methodology consists in the control of seed hydration using low water potential of an active osmotic solutions (osmopriming) or water soaking (hydropriming) [200,201,202]. It has been reported that the application of exogenous melatonin to seeds as a pre-sowing treatment improved seed germination as well as seedling growth and vigour. For example, Małgorzata and colleagues, by treating *Zea mays* seeds with melatonin applied in osmopriming at concentrations of 25–50 µM, observed an acceleration in seed germination along with an increased fresh weight of the seedlings with respect to both control and hydroprimed seeds [203]. Similar results were observed also for melatonin applied in hydropriming on *Cucumis sativus* [204] and *Brassica oleracea rubrum* seeds [186]. However, the beneficial effects derived from melatonin priming treatment as previously discussed were more remarkable during the germination tests performed under suboptimal temperature conditions (10–15 °C) or under heavy metal contamination. In this case, the seedlings grown from seeds treated with melatonin better tolerated abiotic stresses compared to control, also showing an highest germination percentage, seedling weight, chlorophyll content, and phenolic synthesis [186,204]. On the other hand, it is also known that melatonin can act as pro-oxidant agent in plants. In particular, high melatonin levels (>500µM) may cause oxidative changes in the protein aminoacidic sequence [205], as observed in cucumber seeds that showed a disturbed seed germination and viability [204]. Field experiments conducted with seeds of *Cucumis sativus* L., *Zea mays* L., and *Vigna radiata* L. primed with a low dosage of melatonin showed more developed plants with a foliar senescence delayed, and an higher crop yield compared to control plants [206]. In particular, at harvesting time the plants grown from cucumber seeds osmoprimed with 50 µM melatonin produced a higher number and larger fruits than those osmoprimed without the indolamine and/or completely not-osmoprimed [207]. Similar results were also observed on corn and mung bean plants grown after osmopriming with 50–500 µM melatonin [207,208]. The function multiplicity of melatonin is not restricted just to germination, growth, reproduction, and plant general health, but it is also involved in boosting fruit ripening [209]. In plants, the treatment with melatonin up-regulates the transcripts of ethylene signal transduction-related genes and induces production, perception, and signalling of this hormone, consequently accelerating fruit ripening and softening, along with better pigmentation and flavours, as observed in tomato (*Solanum lycopersicum* L.) plants [210]. Proteomic analysis revealed that melatonin treatment improves the content of ripening-related and anthocyanin increase-related proteins [211].

### 5.3. Melatonin Affects Photosynthetic Efficiency

Melatonin is involved also in the reduction of chlorophyll degradation and in the enhancement of photosynthetic efficiency under abiotic stress by regulating the accumulation and function of key biomolecules, including RuBisCO enzyme, proteins, chlorophylls, and nitrogen-related compounds [212]. Indeed, maize (*Zea mays*) plants treated with exogenous melatonin increased total soluble proteins, nitrogen, and RuBisCO content [213]. In rice, the treatment with melatonin caused a significant reduction in chlorophyll degradation, along with suppression in the transcript levels of senescence-related genes [214]. In apple tree leaves whose photosynthetic capacity was partially inhibited because of drought stress, melatonin was able to improve the efficiency of photosystem II under dark and light conditions, also allowing leaves to maintain a higher capacity for CO_2_ assimilation and stomatal conductance [215]. Similar data were obtained in a study related to water-stressed cucumber seedlings, in which melatonin treatment was observed to reduce chlorophyll degradation, increase photosynthetic rate and activities of ROS-scavenging enzymes, basically reducing the effects of drought [187]. Other positive effects of melatonin application were observed on photosynthetic pigments and machinery of the macroalga *Ulva* spp. and *Chara australis*, which reported an increase in the efficiency of the photosystem II reaction centres [216]. Positive effects were also observed in shoot-tip explants of cherry rootstock, in which in addition to its rhizogenic effects, melatonin slightly enhanced the content of photosynthetic pigments [26].

Studies conducted on grafted *Carya cathayensis* showed that plants grown under severe drought stress displayed a drastic reduction of photosynthetic rate, stomatal conductance, and transpiration rate compared to unstressed plants. Moreover, pre-treated plants with 100 µM melatonin showed less drought negative effects with an average enhancement of 24% in gas exchange parameters [34]. In the same plants, drought stress also negatively affects the maximum quantum efficiency (Fv/Fm) and the electron transport rate (ETR) of photosystem II (PSII), but melatonin pre-treatment was able to significantly improves the performance of PSII by enhancing Fv/Fm and Electron Transport Rate (ETR) [34].

Another commonly observed phenomenon resulting from the exogenous application of melatonin is the enhancement in leaf area, a condition that favours the photosynthetic process, especially under water deficit conditions [34,217]. This process includes the improved maintenance of cell turgor and water balance in mesophyll cells by melatonin, conditions that reflect on stomatal conductance [218,219,220,221], involved in the protection or in the recovery of palisade and spongy tissues under drought stress.

As previously mentioned, melatonin also regulates the metabolites of carbon fixation and the carbon metabolism pathways by modulating the expression of RuBisCO (ribulose bisphosphate carboxylase), FBA (fructose-bisphosphate aldolase), FBP (fructose-1,6-bisphosphatase), RPI (ribose 5-phosphate isomerase), and SEBP (sedoheptulose-1,7-bisphosphatase), with an up-regulation of the related metabolites following melatonin treatment, supporting the overall recovery of photosynthetic efficiency in drought-stressed plants [34,222].

For an efficient photosynthesis functioning, gas exchange is extremely important. The melatonin treatment also affects this process, leading to a better stomatal conductance length and width in pre-treated plants [220,223]. All these phenomena lead to the improvement of the stomatal conductance, gas exchange and photosynthetic capacity [220].

As previously mentioned, abiotic stresses may induce ultrastructural changes in plant structures also due to a disruption of the electron transport chain and cellular and photosynthetic machineries caused by an excessive production of ROS and RNS. This overproduction of reactive species leads to a reduction of the photosynthetic efficiency in plants [224,225]. For example, in barley, melatonin application at different concentrations, slowed dark-induced senescence of leaves, delaying the total loss of chlorophyll compared to control leaves incubated in water. Given to the role in promoting leaf senescence, the concomitant treatment with kinetin or ABA and melatonin on barley leaves showed that the effect on chlorophyll degradation correlated to the two above mentioned hormones was not present when melatonin was employed [226]. Another observation comes from the exogenous application of melatonin on apple (*Malus domestica*) leaves, that showed a delayed dark-induced senescence because of the enhancement of ROS scavenging enzyme activities, while showing a higher level of ascorbic acid and glutathione content compared with those measured in the control leaves [227]. This effect of melatonin is associable with its excellent antioxidant properties and with its action on chlorophyll-degrading enzyme genes as observed by [215]. In their experiment on long-term application of melatonin to one-year-old apple trees under drought conditions, a likewise delayed leaf senescence and a significant reduction in chlorophyll degradation were observed. Moreover, these effects can be also linked to the ability of melatonin to suppress the up-regulation of the senescence marker gene 12 (*SAG12*) and monooxygenase senescence related pheophorbide-a oxygenase (*PaO*), proving that this compound can play a role as a regulating factor in induced foliar senescence [228,229]. On the other hand, melatonin is also involved in the recovery of chlorophyll content, thanks to its capacity to down-regulate the expression of chlorophyllase enzymes (CHLASE) [226] which are up-regulated under drought stress and involved in chlorophyll degradation [34,230].

### 5.4. Melatonin Affects Antioxidant Defence System

All the positive effects that melatonin has on plants are basically related to its antioxidant and ROS scavenger activity, resulting in an anti-stress effect that is also expressed by its ability to up-regulate a wide number of stress response genes including heat stress transcription factor A-3 (*HSFA3*), ABA-induced Wheat Plasma Membrane Polypeptide (*AWPM*), cytochrome c-2 (*CYTC2*), stearoyl-acyl carrier protein desaturase (*SAD*), catalase (*CAT*), ascorbate peroxidase (*APX*), mitogen-activated protein kinase (*MAPK*), basic Leucine Zipper Domain 60 (*bZIP60*), luminal binding protein 2 (*BIP2*), luminal binding protein 3 (*BIP3*), and calnexin 1 (*CNX1*), and to down-regulate the stress-related genes calcium dependent protein kinase 1 (*CDPK1*), mitogen-activated protein kinase 1 (*MAPK1*), thermospermine synthase (*TSPMS*), ethylene-responsive transcription factor 4 (*ERF4*), heath shock protein 80 (*HSP80*), and protein early responsive to dehydration 15 (*ERD15*) [231,232,233,234].

Several studies have proven that the endogenous production of melatonin is a consequence of abiotic stress, suggesting that this molecule protects plant cells from oxidative damage. For example, barley [235] and lupin [174] plants treated with different chemical stressors such as zinc, hydrogen peroxide, or sodium chloride, showed an increase of the endogenous melatonin levels. Furthermore, the same plants treated with exogenous melatonin before being subjected to chemical stress, showed an improved vegetative development and survival compared to the untreated control. Similar results were obtained with pea (*Pisum sativum*) plants [236] and red cabbage seedlings [186] grown in presence of toxic copper concentrations, that showed an improved vegetative development and survival following melatonin application. In this context, the authors hypothesized that melatonin may stabilize biological membranes by triggering the activity of antioxidative enzymes and enhancing the scavenging of harmful ROS and RNS that normally degrade the polyunsaturated fats causing the formation of malondialdehyde (MDA) [219,237,238]. A more specific action mechanism involving the antioxidant machinery was described by Sharma and colleagues, who observed an increase of the antioxidant enzyme activity, including superoxide dismutase (SOD), catalase (CAT), peroxidase (POD), and ascorbate peroxidase (APX) [34]. In particular, they reported a 26.1% increase of the enzymatic activity for CAT, 21.42% for POD, and 8.9% for APX in grafted *Carya cathayensis* plants in comparison to untreated plants. Moreover, they also detected an increase of the gene expression of SOD (+56%), CAT (+85%), APX (+81%), and PAL (+200%) in treated plants [34].

### 5.5. Melatonin Interactions with Plant Hormones

In line with the definition of plant growth regulator and biostimulant, melatonin may influence and promote plant growth in various ways, also through the modulation of the biosynthesis of hormones, including abscisic acid (ABA), brassinosteroids (BRs), cytokinins (CKs), gibberellins (GAs), jasmonic acid (JA), auxins (AUXs) and strigolactones (SLs). For example, it has been reported that the treatment of plants with melatonin negatively regulates ABA production by down-regulating the key biosynthetic gene, *NCED3* (9-cis-epoxycarotenoid dioxygenase 3) and up-regulating the genes related to ABA-catabolism [239,240]. Since ABA is actively involved in the response to water stress, melatonin treatment may help plants to counteract this type of abiotic stress. The drought resistance conferred by melatonin to plants is also due to the up-regulation of the cytokinin gene expression, such as histidine kinases (HKs), histidine phosphotransferases (HPs), and the response regulators (Type-A RRs, Type-B RRs). At the same time, melatonin is able to down-regulate the production of zeatin (ZT), a cytokinin derived from adenine that is mainly produced during biotic stress conditions [34].

On the other hand, melatonin also positively affects the biosynthesis of brassinosteroids (24-EBL) and jasmonic acid (JA) via the induction of various biosynthetic genes [34,241]. These processes result in an improved protection against different typology of abiotic stress in which 24-EBL and JA play a key role [242,243,244,245,246,247,248,249]. Finally, melatonin seems also able to induce ethylene biosynthesis through the up-regulation of 1-aminocyclopropane-1-carboxylate (ACC) synthase [229]. However, this up-regulation occurs in a different way from that carried out by IAA, as observed in a recent study in which RNA-Seq analysis were performed on *Arabidopsis thaliana* plants treated with melatonin [229].

### 5.6. Melatonin Affects Primary and Secondary Metabolism

A metabolomic analysis conducted on grafted *Carya cathayensis* plants showed that severe drought stress mostly down-regulated the metabolite production, in fact 1661 metabolites were up-regulated and 2298 were down-regulated compared to control plants. The same analysis conducted on melatonin pre-treated plants grown under severe drought stress compared to untreated water-stressed plants, showed 1203 up-regulated metabolites and only 271 down-regulated metabolites [34]. Moreover, it was observed as a general trend that the severe drought stress condition down-regulated the primary and secondary metabolism, whereas melatonin treatment resulted in their up-regulation [34]. For example, the carotenoid pathway, that is normally down-regulated under severe drought conditions, is instead up-regulated after plant pre-treatment with melatonin [34]. This result can be related to the ability of melatonin in mediating the up-regulation of the genes involved in the carotenoid biosynthetic pathway, *PSY1* (phytoene synthase1) and *CRTISO* (carotenoid isomerase) [210,222,250,251]. Additionally, the carbon fixation seems to be affected in a similar manner. Indeed, this process is down-regulated in drought-stressed plants, whereas is up-regulated in plants pre-treated with melatonin, due to the ability of this molecule to modulate the expression of key genes encoding for RuBisCO, FBA, FBP, SEBP, and RPI [222]. On the other hand, the sugar metabolism and the phenylpropanoid pathways seem not to be affected [34]. Indeed, in both conditions they are up-regulated. However, some experimental results suggest that melatonin can selectively up-regulate the expression of transcription factors involved in anthocyanin biosynthetic pathway (e.g., *MYB*, *bHLH*, and *WD40*) [252]. In this way, melatonin regulates the polyphenol biosynthesis via ethylene cell signalling, by up-regulating the expression of phenylalanine ammonia-lyase (PAL), the key enzyme of the phenylpropanoid pathway [253].

## 6. Role of Melatonin in Animals

In human physiology, endogenous melatonin exerts a wide range of activities. It is a powerful antioxidant molecule acting as a modulator of several processes such as mood, sleep, body temperature, locomotor activity, circadian rhythms, and immunological regulation [14]. On the other hand, in elderly people, an age-related impairment of nocturnal pineal melatonin synthesis was observed, correlated to a variety of chronopathologies and a generalized deterioration of health [254,255,256]. The protective effects that can be derived from the intake of exogenous melatonin is related to the increase of its basal level and it has been deeply studied over the years. The main therapeutic purpose of melatonin regards its use as a chronobiotic agent, in the alleviation of jetlag, in the improvement of sleep quality and in the reduction of sleep onset latency. Moreover, other promoting health actions are reported, including antioxidant [257], anti-inflammatory [14], antiaging, neuroprotective [258], and antitumor [259,260] activities, in addition to a protective effect against cardiovascular diseases [261] and diabetes [129]. In particular, melatonin affects carbohydrate and lipid metabolism and its intake was associated with an improvement of the lipid profiles and insulin-sensitivity of many tissues in ob/ob fatty mice [262] or in diet-induced obese mice [263]. Consequently, the supplementation of human diet with additional melatonin or phytomelatonin may be considered a useful strategy to obtain desirable therapeutic effects without negative consequences [264]. Moreover, clinical studies have demonstrated that the oral administration up to 1000 mg melatonin/day, for 30 days did not produce adverse effects [265,266,267,268]. On the other hand, melatonin consumption results in a high bioavailability due to its stability at digestion condition and its rapid absorption in the gastrointestinal tract. Moreover, this indolamine has a half-life of about 20–40 min in blood and it is excreted via urine. The serum melatonin level after intake of plant foods was studied in several animal models. For example, in a murine model, after the intake of germinated kidney beans (*Phaseolus vulgaris* L.) containing 529 ng/g of phytomelatonin, plasmatic melatonin levels were increased by 16% ninety minutes after the administration [269]. Increasing of serum level of melatonin were also observed in animal model after intake of walnuts [147] and grains [165]. Similar results were observed in humans, after the intake of sweet cherries [270,271], plums [272], grape juice [273], beer [271], fruits like orange, pineapple and banana [274,275].

### 6.1. Melatonin in Sleep Disorders

The most important and known effect derived from the administration of exogenous melatonin, via both dietary supplements or foods, is related to the enhancement of sleep-quality and to the induction of sleep due to its key role in animal circadian rhythms [256,276,277]. Many sleep disorders, such as insomnia [278,279], jetlag [280], night shift-work disorder [281], delayed sleep phase syndrome [282], and age-related disturbances in the sleep–wake rhythm [279,283,284,285], have been successfully treated with melatonin. The positive results in the treatment of all these disorders led to notable improvements in “sleep quality” probably related to the melatonin efficacy in promoting and maintaining sleep if not uniform, and it is influenced by the time of administration [286]. For example, in healthy individuals, melatonin showed significant effect on nighttime sleep only when administrated in the early evening [287]. Moreover, several studies have concluded that physiological doses of melatonin have little or none effect on nighttime sleep, whereas high ‘pharmacological’ doses can have hypnotic activity [288].

### 6.2. Melatonin as Antioxidant

Melatonin is considered a powerful antioxidant. It is a scavenger of RNS and ROS, and it was found to be able to up-regulate antioxidant enzymes such as SOD, CAT, GPX, and POX. Its antioxidant properties make melatonin able to (i) protect from lipid peroxidation with stabilization of biological membranes under various oxidizing conditions, such as ionizing radiation, heavy metal toxicity and drug metabolism; (ii) prevent DNA oxidative damage, (iii) prevent protein oxidation and dysfunction [53,100,289,290,291,292,293,294,295,296]. In particular, the ability of melatonin to modulate redox-sensitive targets, whose structure and function can be compromised even by small changes in the cellular redox balance, makes this indolamine active in preserving cellular functions even following exposure to sub-oxidative stimuli [297].

Melatonin also displays an important synergistic action with other antioxidants, including vitamin C, carotenoids (provitamin A), tocopherols (vitamin E) and polyphenolic compounds [47,238,298]. Consequently, the intaking of melatonin is also associated with a strong rise of the serum antioxidant capacity [31,299,300]. The antioxidant properties of melatonin also continue after its metabolism. Indeed, a number of experimental data showed that melatonin can also generate several antioxidant metabolites [42,53,238,301,302].

Although the antioxidant properties of melatonin have been largely discussed in the past, only recently it is highlighted that higher concentrations of this indolamine may also display pro-oxidant effects under specific experimental conditions. For example, at pharmacological concentrations (~1 nM) of melatonin, this indolamine is able to increase the expression of neuronal nitric oxide synthase (nNOS) leading to elevated nitrite and nitrate production [303]. Moreover, despite it has been also observed that melatonin protect liver mitochondria from oxidative damage at a wide range of concentrations (10 nM to 1 mM), it was also reported that this indolamine may induce ROS production in isolated mitochondria from cancer cell lines [291,304,305,306]. However, it must be pointed out that: (i) the pro-oxidant action of melatonin is not correlated with its cytotoxicity; (ii) the pro-oxidant concentration dependent from cell type; (iii) it has been exclusively observed in in vitro cancer cell culture systems [307].

Most likely, the potential pro-oxidant action mechanism of melatonin depends on the interaction with calmodulin. Indeed, despite this intracellular calcium receptor may positively modulate the activities of several antioxidant enzymes via the inactivation of the nuclear RORα melatonin receptor [308], it appears also to mediate the pro-oxidant action of melatonin by involving 5-lipoxygenase (5-LOX) and phospholipase 2A (PLA2) [309].

### 6.3. Melatonin as Geroprotective Agent

During aging, impairments of the melatonin formation are reported, albeit with a considerable interindividual variability, but with a common reduction of its content and its metabolites in the various body districts [310,311,312,313,314,315]. This condition is mainly related to different neurodegenerative disorders, such as Alzheimer’s disease (AD), other forms of senile dementia caused by a progressive deterioration of the suprachiasmatic nucleus [316], neuronal transmission to the pineal gland [255], or to the pineal gland calcification [317,318,319]. Consequently, scientists assumed that melatonin might be beneficial for the treatment of various age-related pathologies or to restoring a normal condition compensating for its lack. Moreover, melatonin has been reported to inhibit the intrinsic apoptotic pathways in age-related neurodegenerative diseases, including Parkinson disease, Huntington disease, amyotrophic lateral sclerosis [320]. For example, in a transgenic mouse model of Alzheimer’s amyloidosis, it was observed that early treatment with melatonin reduced both the oxidative damage and amyloid accumulation by increasing the survival rate [321]. In another murine model, melatonin treatment alleviated behavioral deficits associated with neuronal apoptosis and cholinergic system dysfunction [322]. Finally, some scientific evidences suggested that melatonin administration in AD patients can significantly delay the progression of the disease and decrease brain atrophy [323]. However, AD is usually diagnosed relatively late in the life cycle, and experimental data report no substantial benefits after a later onset of melatonin treatment [324,325]. Most of the neuroprotective properties and anti-aging effects of melatonin seems to be related to the antioxidant activities of this indolamine. Redox active properties can (i) prevent neuronal death by reducing radical induced apoptosis [320], (ii) reduce beta-amyloid-induced lipid peroxidation [326], and (iii) enhance DNA repair capacity [327].

### 6.4. Melatonin in Other Pathological Conditions

Beneficial effects of melatonin intake from plant foods have been proven against a variety of diseases. Hepatoprotection by antioxidant constituents of coffee, including melatonin, have been observed in several animal model liver diseases, including hepatic fibrosis, steatohepatitis, and carbon tetrachloride induced liver cirrhosis [328,329,330]. The hepatoprotective role of coffee seems to be related to the antioxidant properties of its constituent, in particular to the high melatonin content [146,331,332]. On the other hand, an hepatoprotective role has been also demonstrated for melatonin alone [263,333,334,335,336,337]. Finally, a recent study suggested that a general dietary supplementation with melatonin should be considered for preserving the liver physiology [338].

Positive effects of melatonin from plant foods were observed also towards heart health. In particular, in an ischemia-reperfusion injury model it was demonstrated that heart protection by daily moderate consumption of red wine was exerted by the inhibition of the melatonin receptor [339]. Moreover, in a model of pulmonary hypertension, a chronic dietary melatonin supplementation reduced right ventricle hypertrophy, improved ventricular function, reduced plasma oxidative stress, and cardiac interstitial fibrosis [340,341]. Furthermore, it was also reported that high doses of melatonin (20 mg/kg) are able to inhibit apoptosis and liver damage resulting from the oxidative stress caused by several diseases [342]. These results provided strong evidence that melatonin/phytomelatonin supplementation acts as a key player in cardioprotection and provides cardiovascular benefits [340].

A multitude of studies have shown that melatonin can also act as an anticancer agent. Moreover, in several cancers have been reported reduced both melatonin levels and expression of melatonin-receptors [260,343,344,345]. Antiproliferative activity of melatonin has been demonstrated against several tumor cell lines, including breast, colon-rectal, vaginal, endometrial, lung, liver, lymphoma, pancreatic, prostatic, renal, testicular, ovarian, skin and brain cancer cells. The anti-cancer action of melatonin seems to be related to its ability to reduce DNA damage, up-regulate antioxidative enzymes, and to control the expression of certain oncogenes. Moreover, it was reported that melatonin is able to inhibit fatty acid metabolic signaling, via its membrane receptors, leading to the delayed conversion of linoleic acid into 13-hydroxyoctadecadienoic acid, a mitogenic signaling molecule. On the other hand, experimental data reported synergistic effects of melatonin with chemotherapeutic drugs [205,299,346,347,348,349,350]. Finally, several cancers have been associated with low melatonin levels with deficiencies of melatonin-receptors in damaged tissues [260,343,344,345].

A further correlation between melatonin and cancer, is the association between cancer initiation and progression and disturbances in melatonin circadian rhythm with a consequent chrono-disruption. Indeed, shift workers, among which nurses mainly, airplane crews and miners, have a higher prevalence of breast and prostate cancers [351,352,353]. This is also correlated to the suppressed melatonin levels due to light exposure during night (their working time) [354]. Thus, a continuous reduction in melatonin levels might contribute to the probability of the initiation and/or progression of various types of cancer [349]. All these effects are consistent with those previously reported, and in which tumor-bearing rats were perfused with melatonin-rich blood from a human donor (having nighttime levels of melatonin). In this experimentation, an inhibition of the growth of the transplanted tumor was shown, whereas if the same rats were perfused with melatonin-deficient human blood (having daytime levels of melatonin) tumor growth was promoted [355]. The results of this research were also consistent with the International Agency for Cancer Research (IACR), which classifies light at night as a Group 2A carcinogen [356].

Recent evidences on anti-carcinogenic actions of melatonin from plant foods were also reported by Garcia and colleagues. They compared the effects of walnut flour supplementation (7.5 ng melatonin/g dry weight) with those of synthetic melatonin in a murine model of breast cancer. The results provided new insight into the antitumorigenic and immunomodulatory actions of melatonin and specifically of walnut phytomelatonin [357]. However, despite the beneficial effects that phytomelatonin exerts on cancers in vitro and in animal models, the results from human studies are contradictory and sometimes inconclusive, due to differences in both melatonin sources and experimental models making difficult to compare data and obtain trustful results [349].

## 7. Conclusions

Melatonin is an endogenous indolamine produced by several organisms which, in addition to its physiological actions, has displayed a wide range of different bioactivities. The information contained in this review clarifies the chemical characteristics of melatonin and the typical biosynthetic pathways occurring in both animals and plants. Moreover, the main biochemical and biomolecular differences were highlighted. In this review, a meta-analytic approach was carried out in order to identify the main families, species and tissues specialized in the biosynthesis and storage of melatonin in plants. Cluster analysis revealed that 35 families and 131 species can physiologically produce melatonin, and flowers, seeds, and leaves are the main plant tissues able to store this indolamine. Furthermore, the main role of melatonin in plant and animal cells was also described. Finally, this review analyzes the potential benefits derived from the use of exogenous melatonin on both plants and humans. Indeed, the positive effects reported in previously published research and the absence of toxicity at high dosages encourage the use of this indolamine as supplement in both human and plant nutrition.

## Data Availability

No new data were created or analyzed in this study. Data sharing is not applicable to this article.
